# Quantification of Local Warming Trend: A Remote Sensing-Based Approach

**DOI:** 10.1371/journal.pone.0169423

**Published:** 2017-01-10

**Authors:** Khan Rubayet Rahaman, Quazi K. Hassan

**Affiliations:** Department of Geomatics Engineering, University of Calgary, 2500 University Drive NW, Calgary, Alberta, Canada; University of Maryland at College Park, UNITED STATES

## Abstract

Understanding the warming trends at local level is critical; and, the development of relevant adaptation and mitigation policies at those levels are quite challenging. Here, our overall goal was to generate local warming trend map at 1 km spatial resolution by using: (i) Moderate Resolution Imaging Spectroradiometer (MODIS)-based 8-day composite surface temperature data; (ii) weather station-based yearly average air temperature data; and (iii) air temperature normal (i.e., 30 year average) data over the Canadian province of Alberta during the period 1961–2010. Thus, we analysed the station-based air temperature data in generating relationships between air temperature normal and yearly average air temperature in order to facilitate the selection of year-specific MODIS-based surface temperature data. These MODIS data in conjunction with weather station-based air temperature normal data were then used to model local warming trends. We observed that almost 88% areas of the province experienced warming trends (i.e., up to 1.5°C). The study concluded that remote sensing technology could be useful for delineating generic trends associated with local warming.

## Introduction

Climatic regimes are one of the most critical factors in defining the habitat for all living organisms including human beings. In fact, the changes in the climatic conditions have been impacting us most likely since the inception of the earth system. However, the anthropogenic activities (that include industrialization, changes in land use/land cover, and increase of agriculture/food production) have been influencing such climatic changes to a greater extent over the recent several decades (since 1970’s) in particular through the release of excessive amount of greenhouse gases into the atmosphere [1]. One of the major components of the recent climate change is manifested in the warming (i.e., increment in temperature) trends at global to regional to local-levels. Both the global and regional warming trends are relatively well characterized in comparison to the local warming trends. Some example cases include:

- Climate Research Unit at University of East Anglia and Hadley Centre of UK have developed a land air temperature anomaly database known as CRUTEM4 for analysing global and hemisphere-specific anomalies [2–3]. In this case, they employed approximately 5500 point-specific station measurements of air temperature data across the globe; which was also transformed into a gridded dataset with a 5° x 5° spatial resolution. This database has been added with sea surface historical temperature regimes primarily acquired by ships, drifting, and tethered buoys in order to generate a comprehensive global database with a 5° x 5° spatial resolution (i.e., known as HadCRUT4) [4]. Intergovernmental Panel on Climate Change (IPCC) has also used these datasets for depicting the global warming trends since 1850 [5]. It would be worthwhile to note that the generation of spatial extent (i.e., gridded datasets) was based on the unweighted averaging of the available station/location-specific temperature anomalies.
- Fritzsche [6] analysed warming trends at eleven climatic regions in Canada as a whole using 330 station-specific temperature data over the period of 1948–2009; the data was interpolated as the stations were unevenly distributed across the country.
- Menne et al. [7] studied warming trends over the conterminous United States over the period 1980–2008 by using 1218 stations (which were divided into two categories: good and poor exposure sites). The station-specific data were used to calculate anomalies relative to the 1971–2000 station average-values. These anomalies were then interpolated in generating a gridded dataset with a 0.25° x 0.25° spatial resolution.
- Vanderbei [8] conducted a local warming study by using temperature readings from a single undisturbed location at McGuire Air Force Base, around 50 miles far from New York over the period 1955–2010. They developed a least-absolute-deviations (LAD) regression model that robustly extracted a small linear trend from larger seasonal variation. In this study, these local warming trends were compared against the global one, where, around 9000 weather stations’ data were used.
- Mahlstein et al. [9] also conducted a research on emerging local warming signals through point-based data collected at a weather station. They found that the changes did not follow the global trend computed in 5° x 5° grid in the scope of CRUTEMv4 [2] in some specific locations due to having temperature anomalies at local level.

Understanding the warming trends at both global and regional levels are critical; however, the development of relevant adaptation and mitigation policies at those levels are quite challenging. This is due to the fact that: (i) the spatial distribution of required weather stations’ data are usually uneven; and (ii) building consensus among stakeholders is often difficult. On the other hand, at local level, such policy developments are relatively straightforward, as it requires limited number of weather stations’ data and demands small number of stakeholders’ involvement. Thus, it is critical for us to study local warming in order to comprehend the scientific issues, which will play a significant role in developing and implementing both adaptation and mitigation strategies.

In general, the local warming trends are calculated by using long-term air temperature regimes measured at point-based weather station sites. If both quality and quantity of these required air temperature data are sufficient, then the calculated trends are very accurate. However, there are several issues, such as weather stations are: (i) usually installed in the populated areas thus often lack spatial distribution; (ii) having deficiency in maintaining international standards (i.e., World Meteorological Organization guidelines); (iii) having high installation and maintenance costs in particular to the remote areas; and (iv) having lack of continuity in data acquisition and its quality; etc. In order to overcome these issues, one of the most utilized methods is the use of geographic information system (GIS)-based interpolation techniques (e.g., kriging, inverse distance weighing, spline, etc.) [10–13]. These approaches frequently produce different output maps even using the same input datasets [14]. Fortunately, using spatially continuous dataset acquired by satellite-based remote sensing platforms can eliminate the limitation of these interpolation techniques.

**Fig 1:**
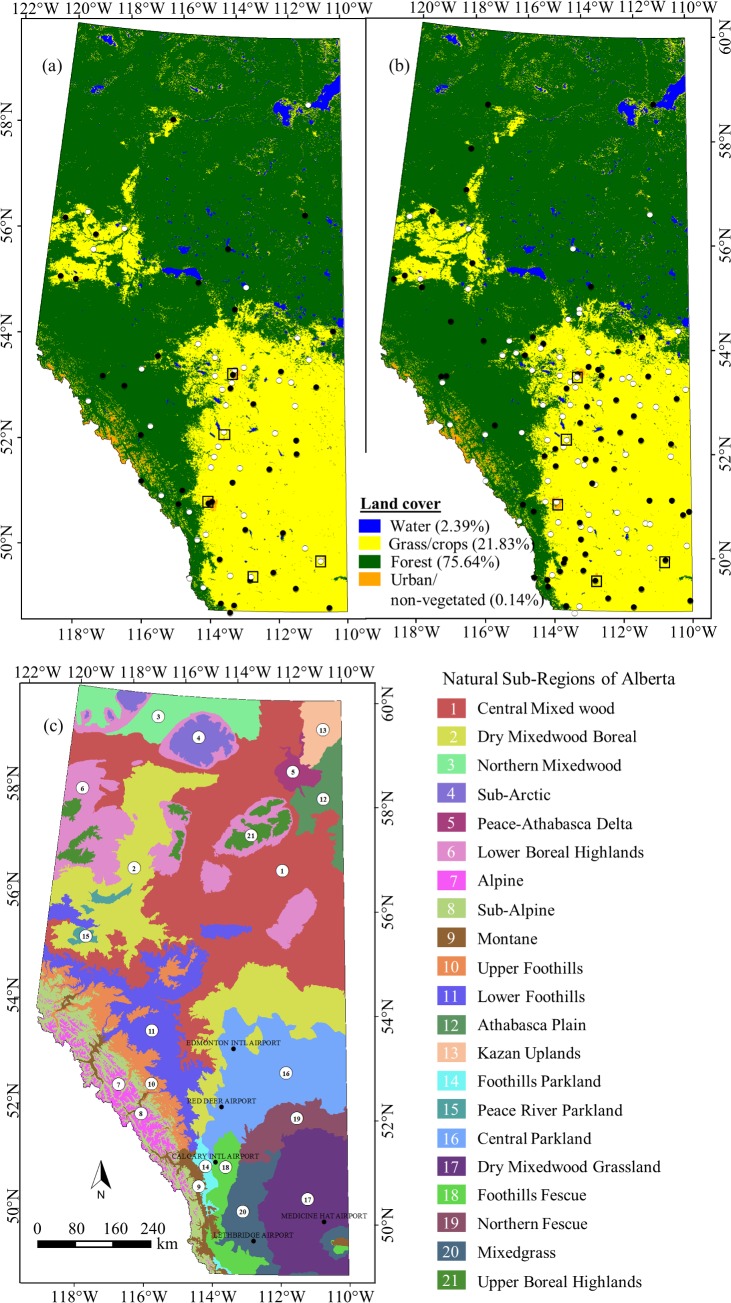
Extent of the study area. Black and white dots (in panels a & b) represented weather stations used for model calibration and validation respectively. The selected stations were based on available ground-based air temperature normal data during 1961–1990 (panel a), and 1981–2010 (panel b). In both panels a & b, the background image representing generic land cover types that was derived from annual composite of MODIS at 500 m spatial resolution. Also the square boxes indicated major 5 city centres within the study area. In addition, panel c showed spatial extent of 21 natural sub-regions within the study area.

Since 1970’s, remote sensing satellites have been operational and acquiring data in the form images. However, its utilization is found to be quite limited in analysing global and regional warming trends [15]. For example, Microwave Sounding Units (MSU) and Advanced Microwave Sounding Units (AMSU) on NOAA satellite data have been analysed during the past several decades (i.e., 1979–2010) in comprehending warming trends over: (i) near global (i.e., ~75°S to 75°N) [16–17]; (ii) tropics (i.e., ~20°S to 20°N) [17–18]; (iii) northern extratropics (i.e., ~20°N to 82.5°N) [17]; and (iv) southern extratropics (i.e., (i.e., ~82.5°S to 20°S) [17]. Despite the importance of these studies, the MSU/AMSU-derived data has limited use in local warming trend analysis because of their coarse spatial resolution (i.e., 2.5° x 2.5°). An alternate may be the employment of thermal remote sensing data that has relatively high spatial resolution in the range ~100–1000 m. These are, in fact, highly used in understanding urban climate (also known as urban heat island) [19–20] rather than local warming trend analysis. Thus, our overall goal was to explore whether it would be possible to comprehend local warming trends over the Canadian province of Alberta over the period 1961–2010 using Moderate Resolution Imaging Spectroradiometer (MODIS)-based surface temperature data at 1 km spatial resolution. Note that contemporary research works also demonstrated are suggesting that the methods of converting air temperature into surface temperature models are providing an average 85% accuracy [21–25]. In order to achieve this, our specific objectives included the: (i) selection of the MODIS-based surface temperature data available over the 2000–2010 that was harmonized with two different air temperature normal regimes (defined as 30 year averages) over the periods 1961–1990 and 1981–2010 acquired at approximately 100 ground-based weather stations (see Fig 1A & 1B for their locations); (ii) development of methods for transforming MODIS-based surface temperature data into air temperature normal at 1 km spatial resolution and its validation; (iii) comprehension of spatial extent of local changes based on the modelled air temperature normal between 1961–1990 and 1981–2010; and (iv) comparison of local warming trends in relation to the regional and global ones.

## Materials and Methods

Here, our study area Alberta (i.e. one of the provinces of Canada), is situated between 49–60°N latitude and 110–120°W longitude (see Fig 1). It exhibits two dominant land cover types, i.e., forest (75.64%) and grass/crops (21.83%). It has a variable topography in the range 162–3,592 m above the mean sea level and having a general increasing trend from north-east to south-west direction. Climatically, the study area experiences relatively cold (i.e., yearly average air temperature varies in the range -3.6 to 1.1°C) and dry (i.e., annual average precipitation varies in the range 377–535 mm) conditions [26]. The study area is partitioned into 21 natural sub-regions on the basis of climatic conditions, soil types, topography, and vegetation types [26]; and shown in Fig 1C. In this study, we used weather/climatic data acquired at point locations available from Environment Canada. These included: (i) yearly average air temperature during the period 2000 to 2010 at the major 5 city centres that comprised of Edmonton, Red Deer, Calgary, Medicine Hat, and Lethbridge (see Fig 1A & 1B for locations); and (ii) air temperature normal during the period 1961–1990 at 76 weather stations (see Fig 1A for locations) and 1981–2010 at 129 stations (see Fig 1B for locations). In addition, we also acquired MODIS-based 8-day composite surface temperature images at 1 km spatial resolution (i.e., MOD11A2 v.005) during the period 2001–2010 available from National Aeronautics and Space Administration (NASA).

**Fig 2:**
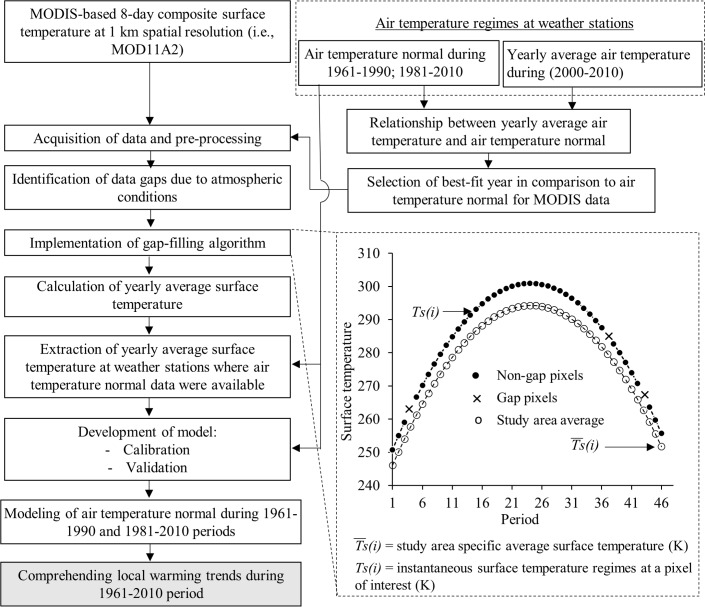
Schematic diagram of the methods employed in this study.

Fig 2 illustrates the methods employed in this study in form of a schematic diagram, which was used to generate local warming trends. The methods consisted of three major components: (i) analysing the station-based air temperature data in generating relationships between air temperature normal and yearly average air temperature to aid the selection of year-specific MODIS-based surface temperature data, (ii) pre-processing of MODIS data and implementing gap filling algorithm, and (iii) modeling of air temperature normals during the periods 1961–1990 and 1981–2010, and generating local warming trends. Brief descriptions of these three steps are summarized in the following sub-sections.

### Analysis of air temperature data and selecting year-specific MODIS-based surface temperature images

In case of analysing air temperature data, we compared the yearly average air temperature for each year over the period 2001–2010 with the air temperature normal during the 1961–1990 and 1981–2010 periods acquired at the 5 major cities (see Fig 1 for location information). Such comparisons were accomplished by generating co-efficient of determination (*r*^*2*^; Eq 1), root mean square error (RMSE; Eq 2), and linear regression analysis. Upon identifying the best-correlated years in relation to the air temperature normal periods, we considered those year-specific MODIS-based surface temperature images best representing the air temperature normal over the period of interest.

$$r^{2}={\left[\frac{\sum \left(T_{a-yearly\;avg.}{-\bar{T_{a}}}_{-yearly\;avg.}\right)(T_{a-normal}{-\bar{T_{a}}}_{-normal})}{\sqrt{\sum {\left(T_{a-yearly\;avg.}{-\bar{T_{a}}}_{-yearly\;avg.}\right)}^{2}}\;\sqrt{\sum {\left(T_{a-normal}{-\bar{T_{a}}}_{-normal}\right)}^{2}}}\right]}^{2}$$ (1)

$$\text{RMSE}=\sqrt{\frac{\sum \;{\left[T_{a-normal}\text{-}T_{a-yearly\;avg.}\right]}^{2}}{n}}$$ (2)

where, *T*_*a-yearly avg*._ and *T*_*a-normal*_ are the yearly average air temperature and air temperature normal; ${\bar{T_{a}}}_{-yearly\;avg.},\;{\bar{T_{a}}}_{-normal}$ are the average values of the yearly average air temperature and air temperature normal; and *n* is the number of observations.

### Pre-processing of MODIS data and implementing gap-filling algorithm

Upon selecting year-specific MODIS-based surface temperature images representing the air temperature normal during the period 1961–1990 and 1981–2010, we found that four panels of data (i.e., four individual images to represent the whole area) were required in order to capture the entire study area. Thus, we prepared mosaic panels and sub-set to the extent of study area. After this part, we generated two datasets each consisting of 46 layers of 8-day composite images. Then, we evaluated the quality of each pixels using quality control information in order to determine the null/gap pixels, which were most likely occurred due to atmospheric conditions like cloud and aerosol loading in particular. In order to eliminate such null pixels, we adopted an algorithm developed by Hassan et al. [21] (see Eqs 3 and 4 for details) so that the surface temperature data in the images could be consistent as a missing data (i.e., gap pixel) might affect the yearly average temperature to a great extent. The algorithm is expressed as follows:

$$A=\frac{\sum_{i=1}^{i=n}\left[{\bar{T}}_{S}(i)-T_{S}(i)\right]}{m}$$ (3)

$$B_{n}={\bar{T}}_{S}(n)-A$$ (4)

where, *Ts(i)* is the instantaneous 8-day composite of surface temperature regimes at a pixel of interest where good quality (i.e., non-impacted by the atmospheric conditions) surface temperature values (with *i* = 1,2,……..n); $\bar{T}s(i)$ is the study area specific average surface temperature upon considering only the good quality pixels for every 8-day period; *n* (= 46) is the total number of 8-day composites, *m* is the total number of good quality 8-day composites, *A* is the average temporal deviation of surface temperature from $\bar{T}s(i)$ over a specific null pixel; and *B*_*n*_ is the estimated surface temperature over the null pixel. Note that we adopted this algorithm as it demonstrated reasonably strong relevance and proven good fit (i.e., *r*^*2*^ in the range 0.83–0.88) in other studies [21–22].

### Modeling air temperature normal and generating local warming trends

After two preceding sub-sections, we calculated yearly average surface temperature for each pixel of interest at 1 km spatial resolution. As a next step, we extracted these yearly average surface temperature values at the selected stations where the ground-based air temperature normal data during the periods 1961–1990 and 1981–2010 were available. In fact, some recent studies transformed such MODIS-derived 8-day composite of surface temperature data into equivalent air temperature regimes at 1 km spatial resolution [21, 23–24, 27]. We, then, divided these data pairs (i.e., yearly average surface temperature vs. air temperature normal) into two sets. The first set consisted of 50% of random data points (known as calibration dataset) that were used to establish relationships between the variables of interest. The derived relationship was applied over the remaining 50% of the yearly average surface temperature data points and then we compared the outcomes against the validation data, i.e., consisting of the unused 50% of the air temperature normal data points. In case such comparisons; we used statistical analysis (i.e., *r*^*2*^ and RMSE; see Eqs 1 and 2) to determine the degree of similarities between the modelled and actual air temperature normal values. Upon evaluation, we used the relationships generated in the process of calibration in order to generate the modeled air temperature maps during the periods 1961–1990 and 1981–2010. Then, we generated a differential map between the air temperature normal maps from the periods 1961–1990 and 1981–2010; and subsequently used to understand local warming trends. Based on secondary sources of information (i.e., World Meteorological Organization; research papers from published documents), our outcomes (i.e., local warming trends) were compared with global [28] and regional model-derived [6, 29] warming trends to quantify their relationships.

As mentioned in the last paragraph, we used 50% ground data for calibration and 50% data for validation. However, we evaluated multiple partitioning of the entire ground-based data, such as 20–80% data for calibration and the remaining for validation in order to find out the optimal combination using the Geospatial Modeling Environment (GEM) [30]. The GEM employed a bivariate uniform random distribution using features space, raster space, or extent in order to define whether a given data-point would fall within a particular rectangular boundary of interest. We, then, evaluated the spatial distribution for all the different partitioning schemas. In this case, we found that the division of 50% was the only one that had revealed the best representation where at least one data point was present for each of the 21 eco-regions across the study area. Finally, we swapped the calibration and validation datasets; and evaluated their performances in comprehending the strength of the data sampling/partitioning strategies.

## Results and Discussion

### Analysis of yearly average air temperature and MODIS-based surface temperature data

**Fig 3:**
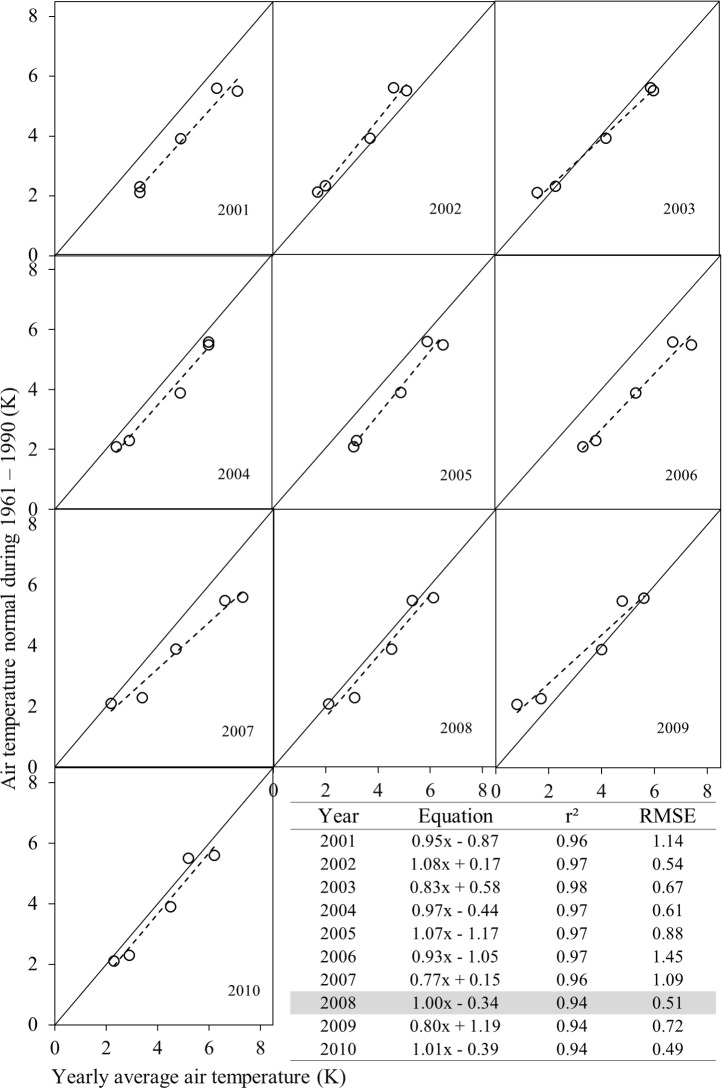
Relationship between yearly average air temperature and air temperature normal 1961–1990; where the solid black line represents 1:1 and black dotted lines represent regression lines.

**Fig 4:**
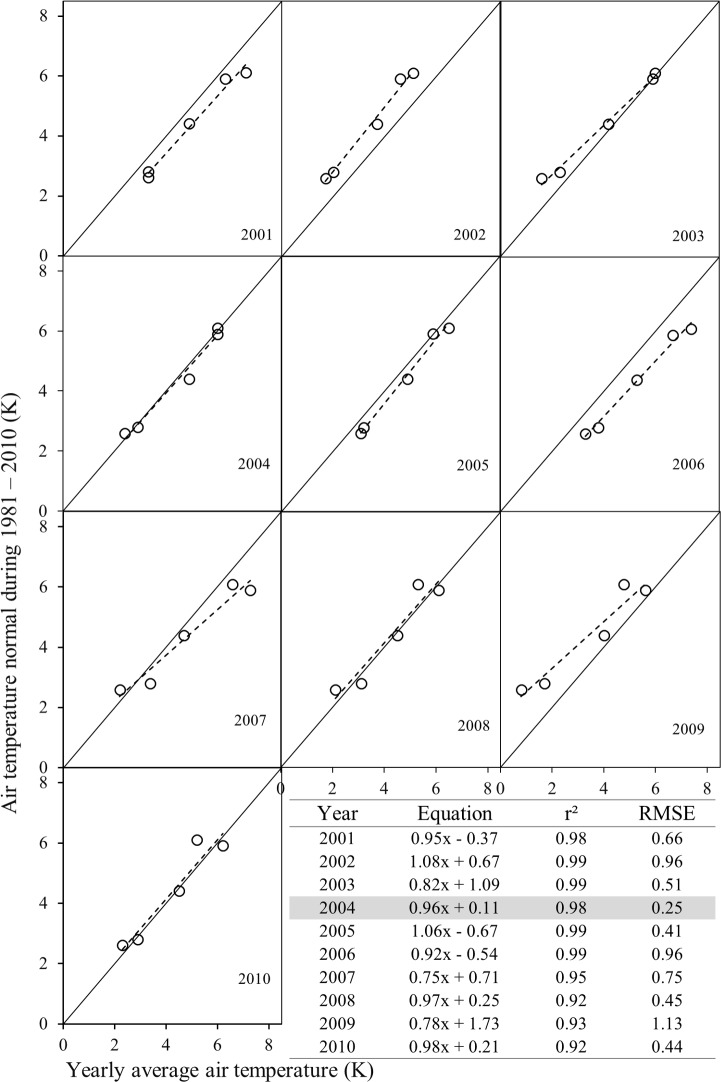
Relationship between yearly average air temperature and air temperature normal 1981–2010; where the solid black line represents 1:1 and black dotted lines represent regression lines.

Figs 3 and 4 show relationships between yearly average air temperature for each of the years over 2001–2010 and air temperature normals during the periods 1961–1990 and 1981–2010 at the five major city centres. Results driven from these relationships illustrated the fact that yearly average air temperature of the year 2008 and 2004 would fit the best with air temperature normals 1916–1990 (i.e., the *r*^*2*^, RMSE, slope, and intercept were 0.94, 0.51°C, 1, and 0.34°C respectively; see Fig 3) and 1981–2010 (i.e., the *r*^*2*^, RMSE, slope, and intercept were 0.98, 0.25°C, 0.96, and 1°C respectively; see Fig 4) respectively. Thus, the years 2004 and 2008 were considered for extracting MODIS-based surface temperature data. However, important question remained in here regarding the choice of 2004 and 2008 yearly average air temperature data to be similar with 1961–1990 and 1981–2010 air temperature normals. Indeed, we considered five weather stations in Alberta which were point-based locations near the airports. The locations (i.e., latitude and longitude) of those weather stations were assumed to be unchanged over time. In addition, over the 30 years’ time period, the surrounding landscapes of weather stations’ did not experience significant changes, as it would not possible to expand settlements and urban facilities at close proximity to the airports. As a result, air temperature normal 1961–1990 and 1981–2010 matched with yearly average air temperature of 2008 and 2004 respectively. It is important to note that around 65% of population of the province of Alberta are living in 5 major city centers (see sub-section “Spatial extent of local warming and its comparison with global and regional inclination” for details). As a result, we have considered these five major cities to imitate yearly average temperature changes with air temperature normals.

### Converting MODIS-based surface temperature into air temperature normal

We analysed 46 layers of MODIS surface temperature data (8-days composite images of the whole year) for each of the years of 2004 and 2005; and found that there were having null pixels (known as gap ones) accounting for around 8% in 2004 and around 5% in 2008. To substitute these null/gap pixels, we implemented gap filling algorithm described in Eqs 3 and 4 and didn’t evaluate its performance as it was a merely adoption of the algorithm from other works [21–23].

**Fig 5:**
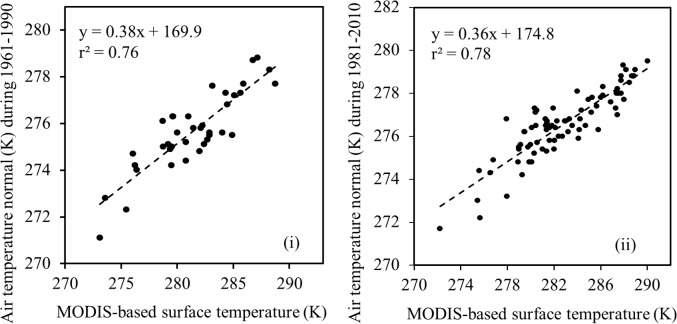
**Establishing relationship between MODIS-based yearly average surface temperature (i.e., from 2008 and 2004) and air temperature normals (i.e., 1961–1990 and 1981–2010) [panel: (i) 2008 vs. 1961–1990, and (ii) 2004 vs. 1981–2010]**.

**Fig 6:**
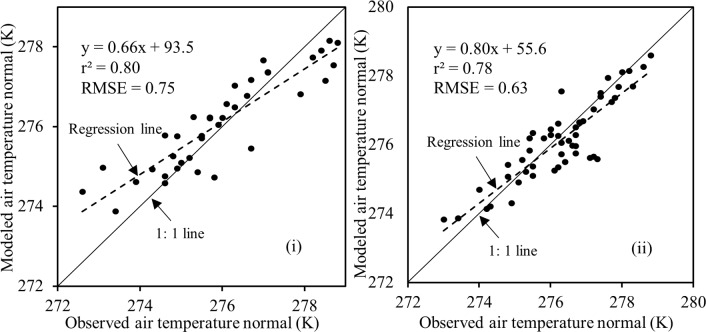
**Validating of MODIS-derived modeled air temperature normal with ground-based (weather stations) air temperature normals [panel: (i) 1961–1990, and (ii) 1981–2010]**.

Fig 5 illustrates the relationships between MODIS-based yearly average surface temperature (i.e., from 2008 and 2004) and air temperature normal (i.e., 2008 vs. 1961–1990 and 2004 vs. 1981–2010). We found that reasonably strong similar relationships were endured (i.e., *r*^*2*^ values were 0.76 and 0.78, for 2008 vs. 1961–1990 and 2004 vs. 1981–2010) between the MODIS-based yearly average surface temperature and air temperature normals. Upon obtaining the relations (i.e., linear regression equations) in Fig 5, we applied them in generating modeled air temperature normal and then compared against the observed ones (see Fig 6). In these cases, we also observed strong similar relations (i.e., *r*^*2*^, and RMSE were in the range 0.78–0.80, and 0.63–0.75 K) were existed between the modeled and observed air temperature normals. As mentioned in the methods, we exchanged the calibration datasets in to validation one and vice-versa; and found indistinguishable results, such as (i) *r*^*2*^ values were 0.76 and 0.78, for 2008 vs. 1961–1990 and 2004 vs. 1981–2010 in the scope of calibration; and (ii) *r*^*2*^, and RMSE were in the range 0.76–0.80, and 0.63–0.67 K in case of validation. Finally, it would be interesting to note that our findings were, in fact, similar to other studies [24, 27]; where, the MODIS-based surface temperature regimes were transformed into equivalent air temperature. However, according to our knowledge, conversion of MODIS-based surface temperature into air temperature normal was not attempted in other studies.

Despite having reasonably strong relations between the modeled and observed air temperature normals, there were about 20% of discrepancies as evident in in Figs 5 and 6. Such discrepancies might exist due to the fact that the air temperature values were acquired at point locations with a footprint of several square meters but the MODIS-based surface temperature values represented averages over 1 x 1 km^2^ area [21]. Also, MODIS-based surface temperature data were acquired between 10.30 AM to 12.00 PM and air temperature normal data was generated by averaging 24 hour measurements over 30 year time period.

### Spatial extent of local warming and its comparison with global and regional inclination

**Fig 7:**
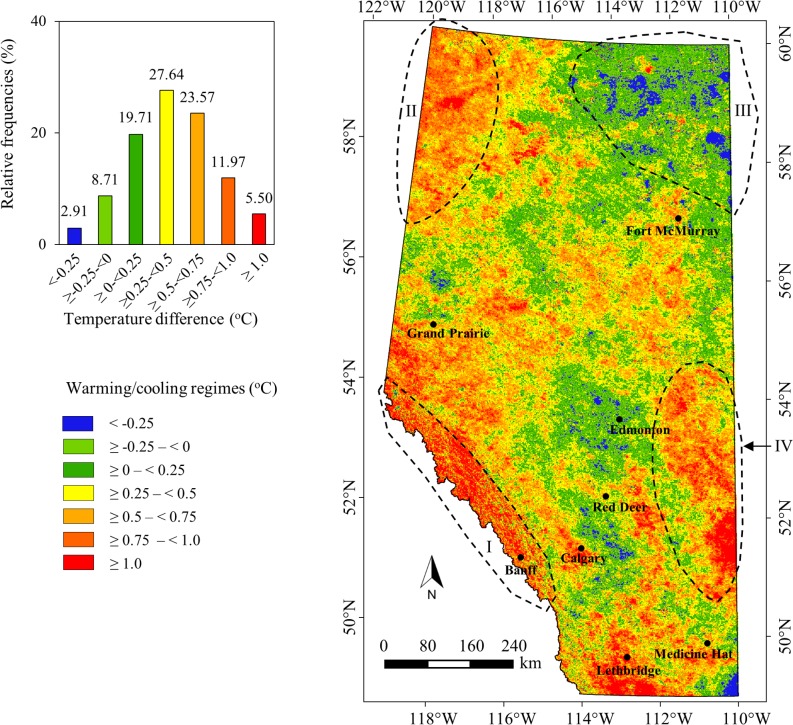
Spatial extent of local warming trends during the period 1961–2010 derived from MODIS-based modeled air temperature normal data.

Fig 7 shows the spatial dynamics of local warming trends over the period 1961–2010; which was derived upon subtracting the two MODIS-derived modeled air temperature normal maps. Our analysis revealed that the local warming trends were observed in most of the areas of the province/study area (i.e., about 88.39%); and generic spatial patterns were summarized as follows:

- About 68% of the areas experienced local warming trends (i.e., from 0.25°C to greater than 1.0°C; see yellow, orange, deep orange, and red color in Fig 7 for details). Also, about 28% of the areas encountered almost no changes (i.e., in the range -0.25°C to +0.25°C; as shown in yellowish green and green color in Fig 7). It would be interesting to note that about 5% areas underwent higher than 1°C warming. On the contrary, an insignificant percentage (i.e., 2.91% in comparison to the remaining 97% of the area) depicted in blue color in Fig 7 demonstrated minor cooling trends (i.e., less then—0.25°C); which we considered as the outliers. These were most likely happened as some of the areas in particular to the north-east portion of the study area (i.e., polygon III in Fig 7) didn’t have enough number of weather stations with long-term data records.
- We observed warming trends (i.e., in the range 0.75°C to more than 1.0°C) in major cities, such as Lethbridge, Medicine Hat, Calgary, Red Deer, Grand Prairie, and Fort McMurray. However, Edmonton, one of the other major cities experienced relatively moderate warming regimes (i.e., ~ 0.25°C). The relatively high warming trends might be associated with the rapid urbanization in the major city centers to accommodate more people and due to expansion of urban infrastructure and road networks [31].
- In Rocky Mountain areas (including Banff National Park, polygon I in Fig 7), it was discerned that warming trends were significantly higher (i.e., 0.75°C to more than 1.0°C). This might happen due to rapid expansion of infrastructure networks to accelerate tourism industries, and because of the environmental changes due to growing number of visitation of tourists every year [32].
- In the North West part of the study area (i.e., polygon II in Fig 7) warming trends were also found to be incremental (i.e., more than 0.75°C). It might happen due to change of landscape composition over the time period and expansion of human activities.
- In the South West part (i.e., polygon IV in Fig 7), warming trends were observed significantly high (i.e., more than 0.75°C). This area had been characterized as western prairie and had been experiencing the cumulative effects of drought, agriculture-based industrialization and catchment modification [33] that might have resulted such locally felt warming trends.

In addition to qualitative analysis of local warming trends as discussed above, we also conducted a quantitative evaluation of local warming trends over the natural regions and sub-regions within the study area (see Table 1 for details, and Fig 1C for spatial extent of natural sub-regions). It was evident that the average local warming trends over the natural sub-regions were in the range -1 (i.e., over Kazan Upland; part of polygon III in Fig 7) to +0.93°C (i.e., over Alpine) with a standard deviation between ± 0.25°C to ± 0.48°C (see Table 1). In order to verify the cooling trends over Kazan Upland sub-region, we strongly felt the need of more ground-based weather station data. Thus, we would recommend others to use this particular cooling trends with extreme caution.

**Table 1:** Warming (change in long-term air temperature) trends over the natural regions and sub-regions of Alberta.

**Natural Regions**	**Natural Sub-regions**	**Average temperature changes (warming trends)** ^**o**^**C**	**Standard deviation ± (**^**o**^**C)**
**Rocky Mountain**	Alpine	0.93	0.42
	Subalpine	0.70	0.39
	Montane	0.50	0.35
**Foothills**	Upper Foothills	0.61	0.26
	Lower Foothills	0.48	0.25
**Grassland**	Dry Mixedgrass	0.49	0.37
	Mixedgrass	0.58	0.39
	Northern Fescue	0.54	0.43
	Foothills Fescue	0.54	0.48
**Parkland**	Foothills Parkland	0.51	0.39
	Central Parkland	0.42	0.38
	Peace River Parkland	0.22	0.29
**Boreal Forest**	Dry Mixedwood	0.40	0.30
	Central Mixedwood	0.32	0.32
	Lower Boreal Highlands	0.44	0.30
	Upper Boreal Highlands	0.35	0.31
	Athabasca Plain	0.02	0.37
	Peace-Athabasca Delta	0.13	0.33
	Northern Mixedwood	0.39	0.39
	Boreal Subarctic	0.27	0.33
**Canada Sheild**	Kazan Upland	-1.00	0.30

In fact, we were unable to compare our findings at natural sub-regional levels due to lack of similar studies. Thus, we also calculated the warming trends over the entire study area, i.e., 0.42 (±0.37)°C during the period 1961–2010; and evaluated against both the global and regional warming trends. For instance: (i) the global warming (that included both land and sea surface temperature) was found to be 0.28°C during the period 1961–2010 [28]; (ii) Canada as a whole had gained an average warming of 1.4°C during the period 1948–2009 [6]; (iii) southern Canada (i.e., up to 60°N latitude) had encountered an average warming between 0.5 to 1.5°C during the period 1900–1998 [29] and (iv) our study area as part of Canadian Prairie provinces had experienced an average regional warming between 0.9 to 1.7°C [6]. While comparing these incremental trends of global and regional warming, we found that the local warming trends derived from MODIS data at 1 km spatial resolution did not follow the same pattern; which, in fact, emphasised on the requirements of more detailed studies about local warming.

Despite demonstrating fairly strong relationships between station-based temperature data and MODIS derived surface temperature data to draw local warming maps, it would be note-worthy to mention the following two issues, such as seasonal variation of temperature and influence of landuse on temperature regimes.

#### Seasonal variation of temperature

In this research, we used MODIS-derived 8-days composite of surface temperature mostly acquired under clear sky condition. However, temperature varied at different scales at diurnal, seasonal, regional, and annual levels. Our intention was to impersonate the RS data (i.e., 8-days MODIS composite data) and station-based climate data. For instance, temperature variations at different scale (i.e., seasonal variation) was not considered as the air temperature normal which mainly considered 30 years’ time period. It would be worthwhile to mention that some of the recent research had used 8-days composite of MODIS-derived surface temperature data to imitate 30 years temperature normal by using solely temperature driven variable (e.g., growing degree days: a favorable temperature regime for plant growth) to convert surface temperature into air temperature [22–23, 25,34]. As a result, we had incorporated the similar ways of extracting remotely sensed data to compare with air temperature normal.

#### Influence of Landuse on temperature regimes

**Fig 8:**
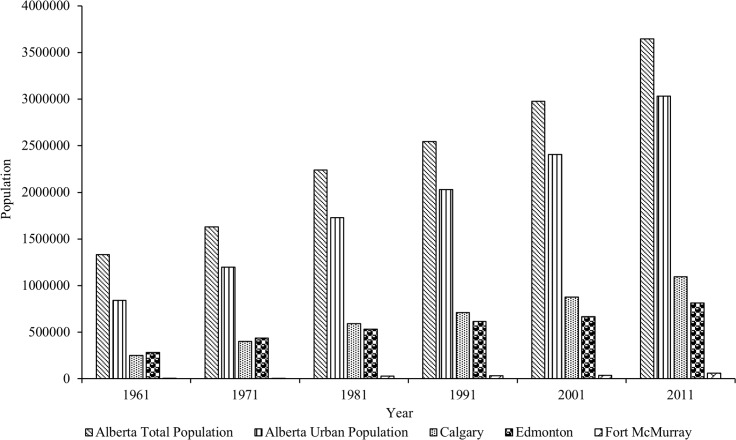
Population growth in Alberta and three fast-paced urbanized cities since 1951 until 2011 [37].

Human activity and landuse practices are cumulatively a major driver of global environmental (i.e., climate) change. Population growth, urban landuse change, and the urban heat island potentials (includes temperature change at local scale) have concurrent relationships for temperature change [35]. Approximately 83% of Albertans live in urban areas, while the remaining 17% live in rural areas [36]. Calgary and Edmonton (two major urban centers in Alberta) regions, along with the corridor that connects the two metropolitan centers, have received the majority of this urbanized population, making it the most urbanized area in the Province. The third-fastest growing region in the Province is in the North, named as the Regional Municipality of Wood Buffalo (this region includes the city of Fort McMurray: the heart of oil sand development) [36]. In fact, Fig 8 shows the above-mentioned rapid growth of urban population in these three major urban centers since 1961. The demands for development pressures to accommodate this growth includes housing, industrial land, water and waste water infrastructure, roads, recreational, and social community provisions are immense. As a result, temperature increases significantly especially in three major urbanized areas (i.e., Calgary, Edmonton, and Fort McMurray; see Fig 7 for details) because of fast-paced landuse change to accommodate rapid development and extension of urban land. Though it is beyond scope of this present study to incorporate land-use component as a matter of temperature change; however, it implies that future research opportunities exist in order to provide higher resolution long-term warming maps over the major urban centers.

## Concluding Remarks

In the scope of this study, we developed a unique model based on remotely sensed MODIS and weather station-based data to evaluate local warming trends in Alberta at 1 km spatial resolution. The proposed methods were consisted of three major steps, such as: (i) developed a relationships of yearly average air temperature and air temperature normal data set to specify MODIS-based surface temperature data; (ii) converted MODIS-based surface temperature data to air temperature normal (i.e., including calibration and validation), and (iii) comprehended spatial extent of local warming based on the modelled air temperature normal between 1961–1990 and 1981–2010. It was found in the study that the average warming trend of Alberta during two climate normals underwent an escalation of 0.42°C. However, some areas experienced relatively cooling trends which were sparsely distributed. It would be important to note that the methods we came across would be useful and applicable to understand local warming trends in other areas if the station based weather data (i.e., yearly average air temperature, and air temperature normal) might available. The study outcome brought a strong message to researchers, professionals, and decision makers that the temperature would be increasing globally but it would not be changing at a similar pattern locally. At the same time, some of the locally affected regions should be taken special care so that the distinct species, forests, and sensitive ecosystems could still survive at the face of local warming trend. Moreover, local governments could now have opportunities to understand more about their own jurisdiction area and the probable warming trend so that they could act quickly to negate potential future risks posed by local warming. Important scope of this study would recommend further extension of the study area to other territories and countries, and to take the challenge of increasing spatial resolution so that the local warming trend could be better understood in compare to global one. However, we would recommend that the method should be evaluated thoroughly prior to apply in other parts of the world.
