# Correction: Quantification of Local Warming Trend: A Remote Sensing-Based Approach

**DOI:** 10.1371/journal.pone.0196882

**Published:** 2018-05-04

**Authors:** Khan Rubayet Rahaman, Quazi K. Hassan, Ehsan H. Chowdhury

Dr. Ehsan H. Chowdhury is not included in the author byline. This author should be listed as the third author and affiliated with The Department of Geomatics Engineering, University of Calgary, 2500 University Drive NW, Calgary, Alberta, Canada. The contributions of this author are as follows: Conceptualization, Data curation, Formal analysis, Funding acquisition, Investigation, Methodology, Validation, Writing—original draft, Writing—review & editing.

The correct citation is: Rahaman KR, Hassan QK, Chowdury EH (2017) Quantification of Local Warming Trend: A Remote Sensing-Based Approach. PLoS ONE 12(1): e0169423. https://doi.org/10.1371/journal.pone.0169423
